# Pulmonary alveolar proteinosis in a cat

**DOI:** 10.1186/s12917-015-0613-4

**Published:** 2015-12-09

**Authors:** Viktor Szatmári, Erik Teske, Peter G. J. Nikkels, Matthias Griese, Pim A. de Jong, Guy Grinwis, Dirk Theegarten, Stefanie Veraa, Frank G. van Steenbeek, Marjolein Drent, Francesco Bonella

**Affiliations:** Department of Clinical Sciences of Companion Animals, Faculty of Veterinary Medicine, Utrecht University, Utrecht, The Netherlands; Department of Pathology, University Medical Centre Utrecht, Utrecht, The Netherlands; Dr von Haunersches Kinderspital, University of Munich, Munich, Germany; Department of Radiology, University Medical Centre Utrecht, Utrecht, The Netherlands; Department of Pathobiology, Faculty of Veterinary Medicine, Utrecht University, Utrecht, The Netherlands; Institute of Pathology and Neuropathology, University Hospital, University of Duisburg-Essen, Essen, Germany; Division of Diagnostic Imaging, Department of Clinical Sciences of Companion Animals, Faculty of Veterinary Medicine, Utrecht University, Utrecht, The Netherlands; Interstitial Lung Diseases, Department of Pharmacology and Toxicology, Faculty of Health, Medicine and Life Science, University Maastricht, Maastricht, The Netherlands; Interstitial and Rare Lung Disease Unit, Ruhrlandklinik, University Hospital, University of Duisburg-Essen, Essen, Germany

**Keywords:** Bronchoalveolar lavage, Cat, Computed tomography scan, Congenital, Electron microscopy, Energy dispersive X-ray analysis, Granulocyte-macrophage colony-stimulating factor, Lung, Respiratory distress, Surfactant

## Abstract

**Background:**

Pulmonary alveolar proteinosis is an extremely rare lung disease in animals and humans. It is characterized by the deposition of a large amount of phospholipoproteinaceous material in the alveoli. There are several possible etiologies, both congenital and acquired. Alveolar macrophages play an important role in the clearance of surfactant. This is the first report of pulmonary alveolar proteinosis in the feline species.

**Case presentation:**

Pulmonary alveolar proteinosis was diagnosed in an 8-month-old cat with chronic tachypnea, failure to thrive and finally respiratory distress. The diagnosis was based on the milky appearance of the bronchoalveolar lavage fluid taken under general anesthesia after bronchoscopy. Because of the worsening respiratory distress and development of anorexia the kitten was euthanized. Histopathology of the lungs showed alveoli and bronchi filled with eosinophilic material. Electron microscopy revealed lamellated intra-alveolar bodies. As the granulocyte-macrophage colony-stimulating factor was elevated in the serum and no autoantibodies against granulocyte-macrophage colony-stimulating factor were detected, a primary hereditary pulmonary alveolar proteinosis was suspected. The underlying cause was thought to be a dysfunction of the receptor of the granulocyte-macrophage colony-stimulating factor, however, a mutation in the genes encoding the alpha and beta chains of this receptor has not been found.

**Conclusion:**

This is the first description of pulmonary alveolar protienosis in a cat. This kitten is thought to have a primary hereditary pulmonary alveolar proteinosis with a possible defect in the signalling pathway of the receptor of the granulocyte-macrophage colony-stimulating factor. The imaging and pathologic findings are similar to those of humans.

## Background

Pulmonary alveolar proteinosis (PAP) is a rare lung disease in animals and humans. It is a heterogeneous group of conditions and characterized by deposition of a large amount of phospholipoproteinaceous material in the alveoli [1]. The incidence in humans is up to 6 cases per 1,000,000 people [1]. Though there are a few canine case reports of PAP [2–4], to the best of our knowledge PAP has not been described so far in the cat [5].

The main pathogenic mechanism of PAP is the insufficient clearance of the surfactant in the alveolar space, leading to its accumulation and consequent respiratory insufficiency [6]. Surfactant is a surface-active lipoprotein complex that prevents alveolar collapse. It contains mostly phospholipids and 10 % proteins including surfactant protein A, B, C and D [6]. Surfactant is produced by the alveolar type II epithelial cells, which are also responsible for its clearance together with alveolar macrophages [6, 7]. According to the most recent classification in human beings, PAP can be either primary or secondary [1]. In addition to these groups a third group, the so called PAP-like diseases, has been described. Within the primary group, two forms have been identified: autoimmune and hereditary. Primary autoimmune PAP is characterized by the presence of antibodies against granulocyte-macrophage colony-stimulating factor (GM-CSF) [7–9]. GM-CSF is essential for the differentiation and activation of alveolar macrophages and for the maintenance of surfactant homeostasis [6, 7, 10, 11]. Deficient alveolar macrophages lead to a reduced surfactant clearance. Primary hereditary PAP is due to gene mutations involving the beta or more rarely alpha chain of GM-CSF receptor, leading to the impairment of GM-CSF signaling [1, 10]. Secondary PAP is probably caused by factors that reduce the number or the function of alveolar macrophages [1]. There are several conditions that can lead to secondary PAP in humans, such as inhalation exposure (e.g. silicon, cement, bakery flour, cleaning products), infectious diseases (such as HIV), amyloidosis, hematologic disorders (such as leukemias), other malignancies (such as adenocarcinoma), and various other disorders (such as lysinuric protein intolerance, severe combined immunodeficiency) [1, 12]. PAP-like diseases are due to genetic mutations that encode surfactant protein-B (SP-B) or SP-C leading to surfactant deficiency or abnormal production (oligomers and dimers), or a mutation of the gene encoding the membrane lipid transporter ABCA3 on type II pneumocytes, with accumulation of abnormal lamellar bodies [1, 10, 13–17]. The principal consequence is the gross distortion of lung structure due to widening of alveolar walls and extensive fibrosis [10]. Macrophages are not primarily involved in the pathogenesis of PAP-like diseases; they appear foamy as the result of intra-alveolar accumulation of abnormal overproduced surfactant sub-forms (oligomers and dimers). This is in contrast to autoimmune PAP where surfactant is structurally and functionally normal but macrophages do not work properly. It is thought that abnormal surfactant (oligomers and dimers) cannot be completely digested by alveolar macrophages, but there is no clear evidence to prove this.

## Case presentation

An 8-month-old female domestic shorthair kitten was referred to the Companion Animal Clinic of the Faculty of Veterinary Medicine of the Utrecht University because of chronic shortness of breath. Tachypnea and forced breathing was noticed since 3 months of age. This was the smallest kitten in the litter of four. The queen was imported from Morocco and was living in the Netherlands. The father was an unknown, presumably Dutch, stray cat. The queen and also her mother have always been clinically healthy. The presented kitten originated from the third litter of the queen.

Besides the slowly worsening respiratory distress the kitten developed a poor appetite and severe exercise intolerance. No cough or other clinical signs (including fever or signs of recurrent infections) were noticed. The kitten was living in a city center. The owner did not have any known potentially hazardous hobbies (such as painting). No possible exposure to inhalational environmental agents was identified during multiple interviews with the owner. Vaccinations and deworming were up-to-date.

At presentation the kitten was depressed, weak and showed tachypnea (60 breaths/min at rest) with a prolonged inspiration. The kitten was too small for its age and had a poorly kept hair coat and poor body condition, weighing 2.5 kg (5.5 lbs). Occasional wheezes were heard. Lung auscultation revealed increased respiratory sounds. The rest of the physical examination was unremarkable.

**Fig. 1:**
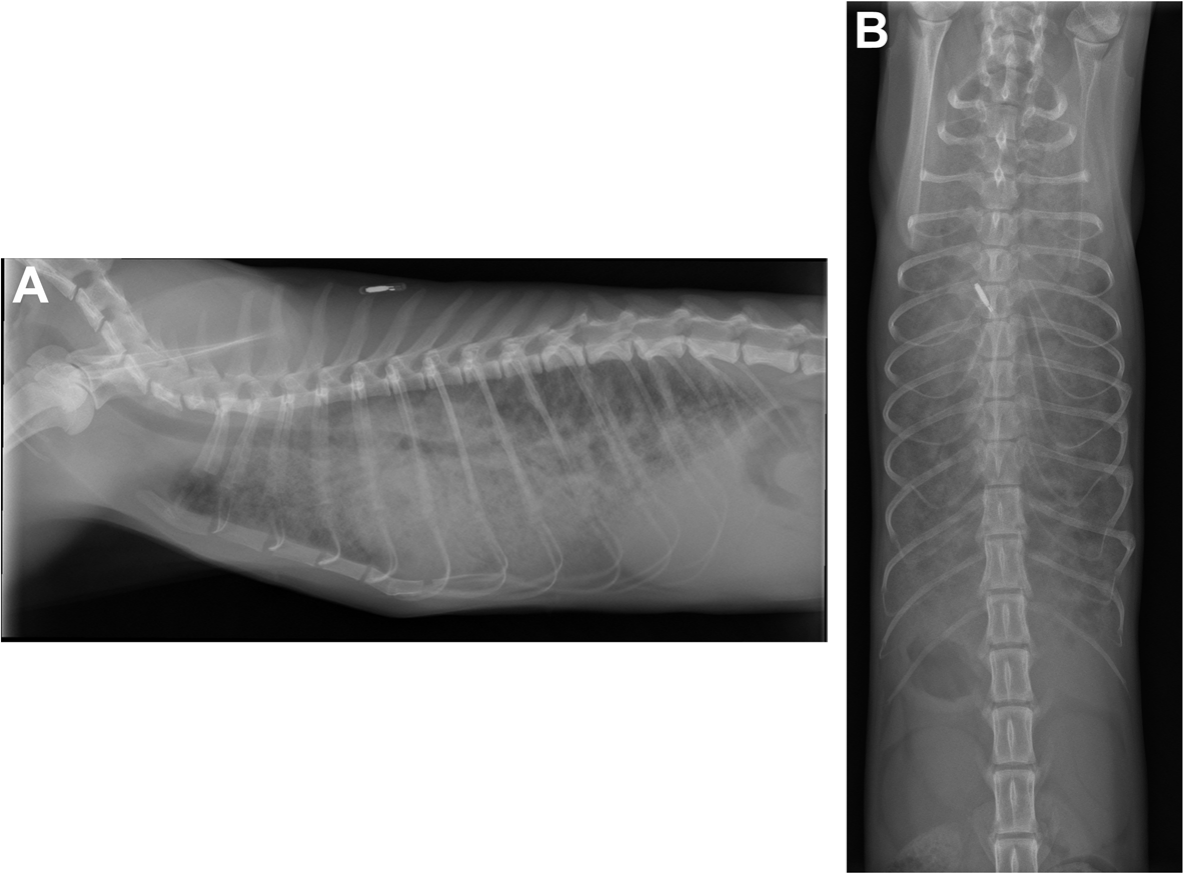
Thoracic radiographs show diffuse patchy alveolar and interstitial lung pattern. **a** Left lateral projection **b** Dorso-ventral projection

Thoracic radiographs showed a severe generalized interstitial and alveolar pattern in all lung lobes (Fig. 1). These findings were not found to be characteristic of any reported pulmonary condition in the cat. Fecal examination (Baermann larva isolation and flotation on 3 samples) was performed to rule out lung worm infection, which was negative. Hematologic and coagulation blood tests (i.e. erythrocyte, leukocyte and platelet counts, fibrinogen concentration, prothrombin time, activated partial thromboplastin time) showed no abnormalities. Snap tests for Feline Leukemia Virus and Feline Immunodeficiency Virus were negative. Echocardiogram did not show any abnormalities.

To further characterize the pulmonary disease a bronchoscopy was performed. The kitten was pretreated with subcutaneous terbutaline to prevent procedure-related bronchoconstriction as recommended by Johnson & Vernau [18]. After premedication with acepromazine (50 μg/cat), methadone (500 μg/cat) and glycopyrrolate (25 μg/cat), general anesthesia was induced and maintained with intravenous propofol (0.15 mg/kg/min). Prior to induction of anesthesia oxygenation was performed by oxygen flow by. During the procedure oxygen was administered through the bronchoscope in a rate of 4 l/min. The kitten was breathing spontaneously and was not intubated, therefore also not ventilated during the procedure. Bronchoscopy with a 3-mm rigid scope did not show any obvious abnormalities, except when the instrument was pulled back, the bronchoscope retracted a thin whitish membrane from the bronchial wall. After completing the bronchoscopy a blind bronchoalveolar lavage was taken by injecting 5 ml of physiologic NaCl solution into a tertiary bronchus through an end-hole canine urinary catheter (50 cm with 2 mm outer diameter). Despite of oxygen enrichment the hemoglobin-saturation was never above 85 % during the bronchoscopy. After taking the bronchoalveolar lavage no expiratory CO_2_ was detected for about 90 s. The recovery from the anesthesia was prolonged, which made hospitalization necessary in an oxygen cage. An arterial blood gas analysis (when 40 % oxygen was inhaled) showed a severe hypoxemia (PO_2_ 5.96 kPa). The next day the kitten’s clinical condition was comparable to the preanesthetic state and was discharged from the hospital. No medication was prescribed.

**Fig. 2:**
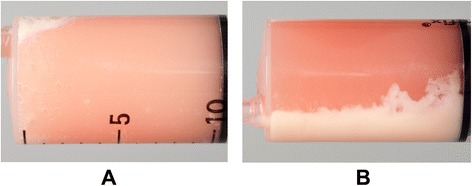
Bronchoalveolar lavage fluid. **a** Macroscopic milky appearance. **b** After a couple of hours a clear separation of milky sediment and supernatant was evident

**Fig. 3:**
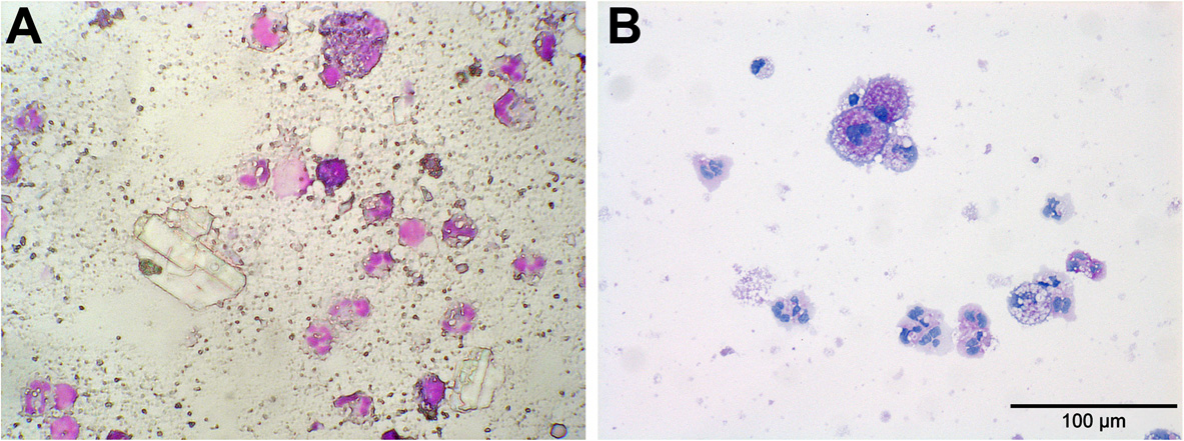
Cytology of the bronchoalveolar lavage fluid. **a** Increased number of neutrophil granulocytes and macrophages. In addition to the increased cellular components there are several crystals and cell debris. Hemacolor stain. **b** The alveolar macrophages have a foamy aspect and are filled with PAS-positive material

The bronchoalveolar lavage fluid (BAL) had a milky appearance (Fig. 2). Cytology of the BAL showed a large amount of crystals and subjectively increased numbers of macrophages and neutrophil granulocytes (Fig. 3). Bacterial culture was negative. The macrophages contained phagocytized particles, which stained positive with the periodic acid Schiff (PAS) stain (Fig. 3).

Based on the results of the above described investigations pulmonary alveolar proteinosis (PAP) was suspected [1, 2]. As this kitten had experienced respiratory problems since a very young age, a congenital anomaly was suspected. Because alveolar proteinosis has not yet been reported in the feline species and only a few case reports were found in the literature about dogs at the time of the clinical presentation [2–5], besides a thorough literature search, a human pediatric pulmonologist was consulted so as to look for potentially curable underlying diseases. Therapeutic lung lavage, which is the treatment of choice in humans with alveolar proteinosis [1, 12, 19], was considered to be a technically challenging procedure with an unacceptably high risk and presumably fatal outcome, as the kitten almost died during the diagnostic bronchial wash.

A quantitative amino acid column chromatography was made from a plasma sample to look for metabolic diseases such as lysinuric protein intolerance, which is reported to be a rare underlying cause of alveolar pulmonary proteinosis, especially in Finnish children [1, 12]. This test showed no abnormalities in the concentrations of the following amino acids: taurine, asparagine acid, hydroxyl-proline, serine, asparagine, glutamic acid, glutamine, proline, glycine, alanine, citrulline, alpha-amino butyric acid, valine, cystine, methionine, isoleucine, leucine, tyrosine, phenylalanine, ornithine, histidine, lysine and arginine. The plasma lysine concentration was not decreased, and the urinary lysine concentration was not elevated either. As no reference ranges were known for the cat at the laboratory, the plasma samples of 3 other cats were compared to the patient’s sample. No obvious differences were detected between the values of the patient and the controls.

Because of the progressively worsening respiratory distress and the development of complete anorexia as well as the poor prognosis the owner decided to have the kitten euthanized one month after the bronchoscopy, at 9 months of age.

**Fig. 4:**
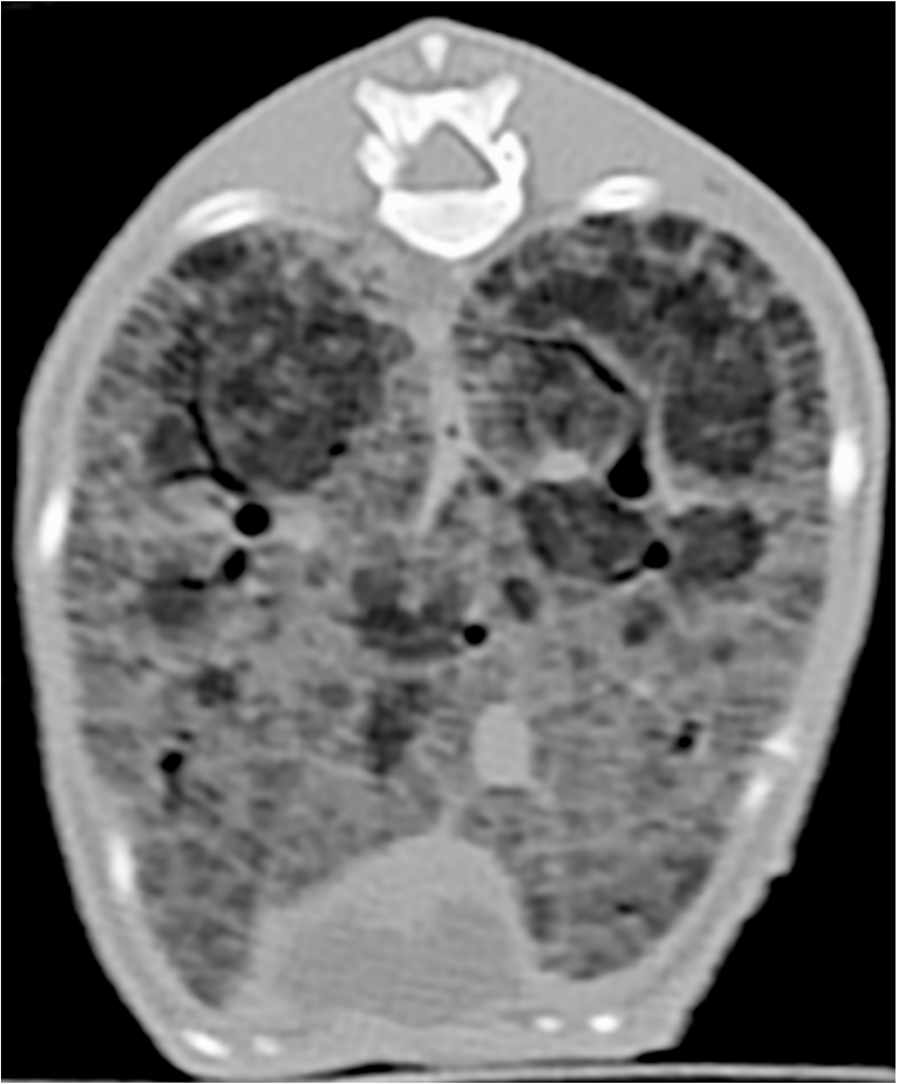
Computed tomography (CT) scan of the lung shows areas of ground glass opacity and interlobular septal thickening along with normal areas. This pattern is also known as crazy paving

Immediately after euthanasia a postmortem computed tomography (CT-scan) of the thorax was performed with an informed consent from the owner. After intra-tracheal intubation the lungs were inflated with 10 mmHg positive pressure. The CT-scan showed diffuse ground glass opacity and thickened intra- and interlobular septa with some localized normal looking regions (Fig. 4). This finding is described in the human literature as crazy paving pattern [20].

**Fig. 5:**
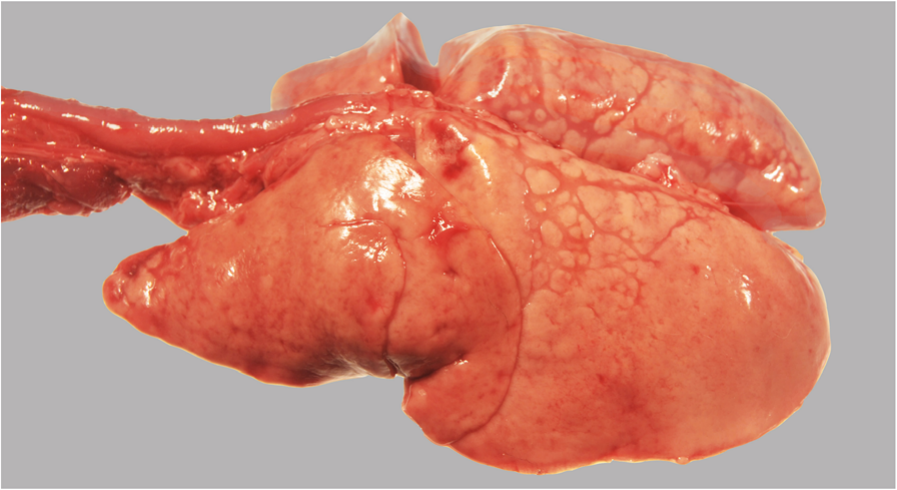
Necropsy shows a diffusely pale, poorly collapsed and firm lung

The owner gave permission for a post mortem examination. Necropsy revealed generalized abnormalities in all lung lobes. The lungs were whitish, not collapsed and firm on palpation (Fig. 5). The cut surfaces were dry. Other organs looked macroscopically normal.

**Fig. 6:**
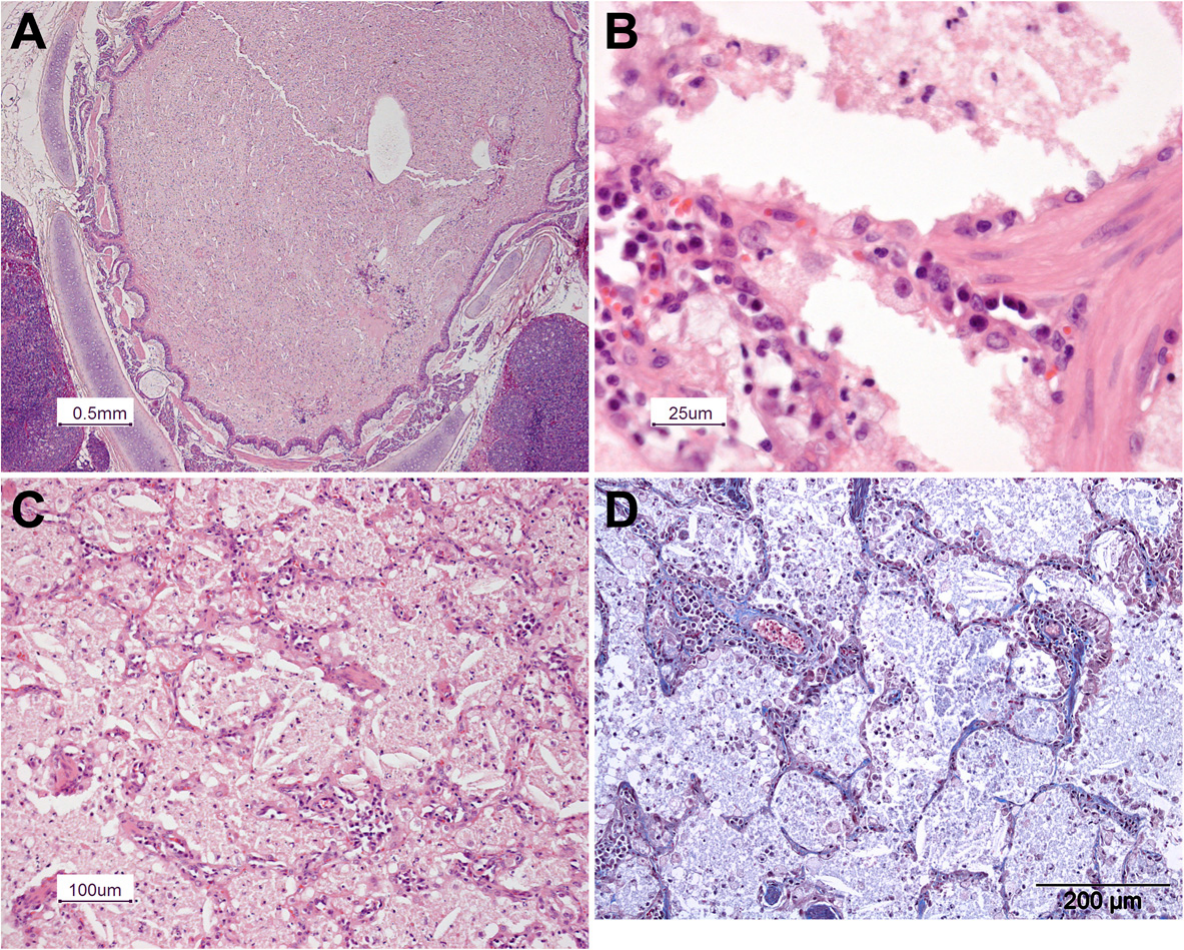
Photomicrographs of the lung. **a** The alveoli and bronchi are filled with eosinophilic material. Hematoxilin & eosin staining. **b** The cuboidal type II alveolar epithelial cells have a foamy appearance. Hematoxilin & eosin staining. **c** In the eosinophilic material crystals can be recognized. Hematoxilin & eosin staining. **d** No extensive fibrous tissue present. Masson’s trichrome staining

Histopathology showed that the alveoli, bronchi and bronchioli were filled with eosinophilic fine-granular fluid and cholesterol clefts (Fig. 6). The eosinophilic material stained positive with PAS. Cholesterol crystals were not found in the pulmonary interstitium. There was a moderately increased number of alveolar macrophages, all with foamy cytoplasm. The alveolar walls were thickened, which was caused by the proliferation of type II pneumocytes as well as infiltration with lymphocytes and plasma cells. The pleura was thickened due to fibrosis. No marked increase in fibrous tissue in the interstitium of the lungs was noted when stained with the Masson’s trichrome (Fig. 6). Histologically the liver revealed focal centrilobular necrosis of hepatocytes, possibly due to hypoxia, and moderate hyperemia. The brain did not show significant morphological changes.

**Fig. 7:**
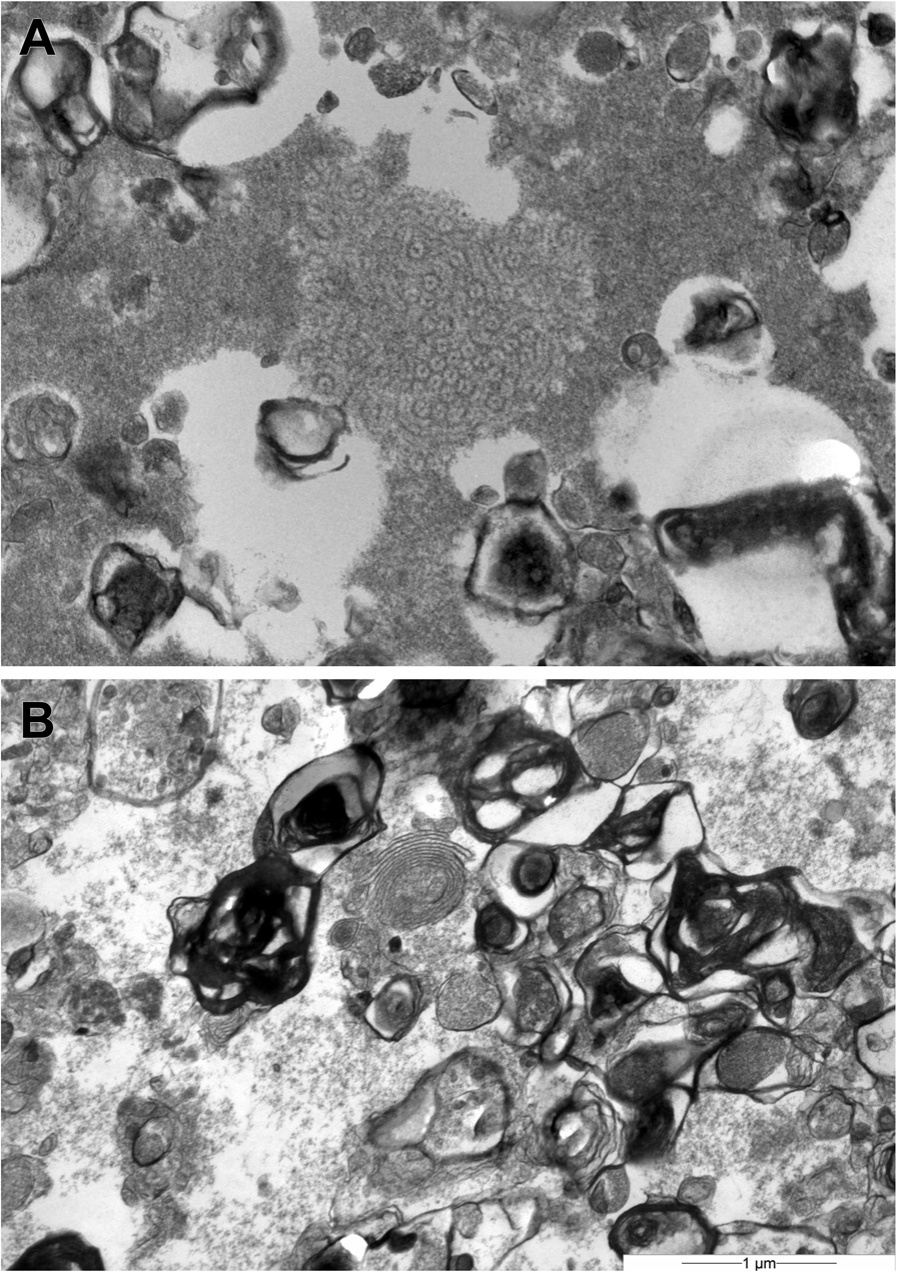
Transmission electron microscopy of the content of the alveoli. **a** Amorphous material and the presence of crystals can be appreciated. **b** Multilamellated structures, compatible with abnormal surfactant forms. 22000 × magnification

**Fig. 8:**
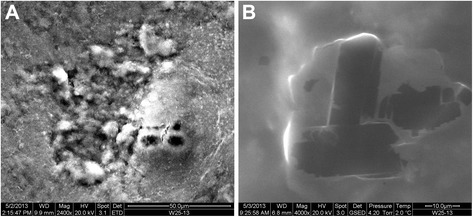
Scanning electron microscopic image of the alveolar content. **a** Negative backscattered electron image obtained with the scanning electron microscope in an area of proteinosis shows dark particles within the respiratory size range, ie less than 5 nm (magnification × 2400). **b** At higher magnification (x4000) the black particles appear angulated and fragmented

**Fig. 9:**
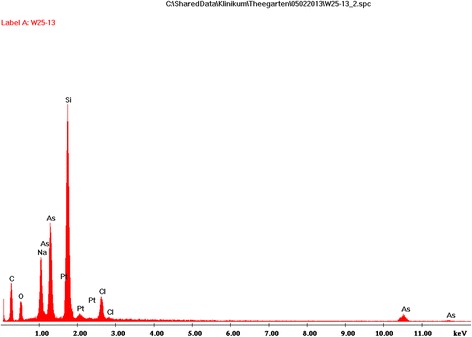
Energy dispersive X-ray analysis of the alveolar content. Energy dispersive X-ray analysis of several particles (see Fig. 8) identified two principal elements: silicon (Si) and arsenic (As). A representative energy dispersive spectrum from one of these particles shows a high peak for silicon and multiple peaks for arsenic (including the small peak at 10.500 keV). Other elements were not always present in the particles

Transmission electron microscopy of the intra-alveolar eosinophilic material showed abnormal lamellar bodies (Fig. 7). To identify the particles within the proteinaceous intra-alveolar material scanning electron microscopy and energy dispersive X-ray analysis (EDS) were performed on the BAL. These techniques detected the presence of silicon (Si) and arsenic (As). Details are shown in Figs. 8 and 9.

Although all the above-described findings confirm the diagnosis of pulmonary alveolar proteinosis [11], this is a heterogeneous group of disorders with several possible etiologies [1, 8]. To look for the possible cause a number of additional investigations were performed from the stored blood and BAL samples.

No autoantibodies against GM-CSF were found in the kitten’s plasma. Its concentration was less than 0.156 μg/ml (normal level in humans is less than 3 μg/ml). Because an assay for feline species was not commercially available, a human assay was used [21]. Serum from an adult cat without respiratory signs was used as a negative control. No feline positive control is available.

GM-CSF was measured in the BAL (3.1 pg/ml) and in the serum (2.8 pg/ml) of the affected kitten. The GM-CSF concentration was also measured in the serum samples of 14 control cats, and was found to be less than 1 pg/ml in each of them.

A genetic analysis was performed to pinpoint a possible mutation in the gene coding the alpha and beta chains of the GM-CSF receptor (CSF2RA and CSF2RB). Primers were designed using PerlPrimer v1.1.14 on the Felis_catus-6.2 assembly. DNA sequence analysis was performed on the exons including the splice sites of CSF2RA and CSF2RB in the affected and 3 healthy cats. Detected variations which were found in the affected cat were validated in a replication cohort of 42 healthy cats. DNA sequencing of CSF2RA did not reveal differences between the affected and the healthy cats that could have caused the disorder (Table 1). The only coding variant detected showed the wild type allele in the affected cat. In CSF2RB two coding variants were detected. The A652G variant was found to be heterozygous in the affected cat and homozygous wild type in the initial 3 controls. The G2674A variant, which appeared to be the most interesting because its genotype fitted the recessive model the best, was found to be wild type in the 3 unaffected cats, but mutant in the patient. Both variants were verified in the larger cohort of healthy cats but appeared to be abundantly present in the cat population.

**Table 1:** Detected coding variants in the DNA of the alpha and beta chains of the Granulocyte-Macrophage Colony - Stimulating Factor receptor (CSF2RA and CSF2RB)

gene	mRNA change	codon change	affected	unaffecteds	healthy population
CSF2RA	G282A	V95M	G	R,R,R	NA
CSF2RB	A652G	H218R	R	A,A,A	29 % A; 52 % R; 19 % G
CSF2RB	G2674A	E892K	A	G,G,G	34 % A; 7 % R; 59 % G

## Conclusions

In the above-described young cat many potential disorders that may cause PAP have been excluded. Because of the absence of GM-CSF autoantibodies a primary autoimmune PAP can be excluded [7, 8]. The increased concentration of GM-CSF in the serum is compatible with a dysfunction of the GM-CSF receptor [8]. Therefore, the genes encoding the alpha and beta chains of the GM-CSF receptor, and not those encoding SP or ABCA3, have been investigated. No mutation has been found that can lead to a dysfunction of this receptor. Because of the presence of intra-alveolar silicon in the BAL, secondary PAP due to silicon (and possibly arsenic) inhalation may also be possible [1, 22, 23]. However, due to the fact that the majority of urban cats in the Western countries come in contact with silicon through silicon-containing litter box filling on a daily basis [24] and so far no PAP has been reported in cats, a secondary PAP in the present kitten is unlikely. On the other hand, data on concentration or presence of silicon and arsenic particles in BAL of healthy cats are lacking, therefore we cannot state that the presence of these particles in the BAL is causative of PAP in this case or even harmful at all. This amount of intra-alveolar silicon may be a “normal” finding in cats in urban environment. The underlying defect which led to PAP in this kitten is thought to be present in the signalling pathway of the GM-CSF receptor.

## Abbreviations

BAL: Bronchoalveolar lavage fluid
CSF2RA and CSF2RB: Alpha and beta chains of the GM-CSF receptor
CT-scan: Computed tomography
EDS: Energy dispersive X-ray analysis
GM-CSF: Granulocyte-macrophage colony-stimulating factor
HIV: Human immunodeficiency virus
PAP: Pulmonary alveolar proteinosis
PAS: Periodic acid Schiff
SP: Surfactant protein
